# Assessing the Impact on Electronic Health Record Burden After Five Years of Physician Engagement in a Canadian Mental Health Organization: Mixed-Methods Study

**DOI:** 10.2196/65656

**Published:** 2025-05-09

**Authors:** Tania Tajirian, Brian Lo, Gillian Strudwick, Adam Tasca, Emily Kendell, Brittany Poynter, Sanjeev Kumar, Po-Yen (Brian) Chang, Candice Kung, Debbie Schachter, Gwyneth Zai, Michael Kiang, Tamara Hoppe, Sara Ling, Uzma Haider, Kavini Rabel, Noelle Coombe, Damian Jankowicz, Sanjeev Sockalingam

**Affiliations:** 1 Department of Family and Community Medicine, University of Toronto, Toronto, ON, Canada; 2 Centre for Addiction and Mental Health, Office 6168G, 100 Stokes Street, Toronto, ON, Canada, 1 (416) 535-8501 ext 30515; 3 Institute for Health Policy, Management and Evaluation, University of Toronto, Toronto, ON, Canada; 4 Information Technology, Unity Health Toronto, Toronto, ON, Canada; 5 Department of Psychiatry, University of Toronto, Toronto, ON, Canada; 6 Lawrence Bloomberg Faculty of Nursing, University of Toronto, Toronto, ON, Canada

**Keywords:** electronic health records, health informatics, documentation burden, physician burnout, engagement strategy, evaluation, EHR, burden, physician, cross-sectional survey

## Abstract

**Background:**

The burden caused by the use of electronic health record (EHR) systems continues to be an important issue for health care organizations, especially given human resource shortages in health care systems globally. As physicians report spending 2 hours documenting for every hour of patient care, there has been strong interest from many organizations to understand and address the root causes of physician burnout due to EHR burden.

**Objective:**

This study focuses on evaluating physician burnout related to EHR usage and the impact of a physician engagement strategy at a Canadian mental health organization 5 years after implementation.

**Methods:**

A cross-sectional survey was conducted to assess the perceived impact of the physician engagement strategy on burnout associated with EHR use. Physicians were invited to participate in a web-based survey that included the Mini-Z Burnout questionnaire, along with questions about their perceptions of the EHR and the effectiveness of the initiatives within the physician engagement strategy. Descriptive statistics were applied to analyze the quantitative data, while thematic analysis was used for the qualitative data.

**Results:**

Of the 254 physicians invited, 128 completed the survey, resulting in a 50% response rate. Among the respondents, 26% (33/128) met the criteria for burnout according to the Mini-Z questionnaire, with 61% (20/33) of these attributing their burnout to EHR use. About 52% of participants indicated that the EHR improves communication (67/128) and 38% agreed that the EHR enables high-quality care (49/128). Regarding the physician engagement strategy initiatives, 39% (50/128) agreed that communication through the strategy is efficient, and 75% (96/128) felt more proficient in using the EHR. However, additional areas for improvement within the EHR were identified, including (1) medication reconciliation and prescription processes; (2) chart navigation and information retrieval; (3) longitudinal medication history; and (4) technology infrastructure challenges.

**Conclusions:**

This study highlights the potential impact of EHRs on physician burnout and the effectiveness of a unique physician engagement strategy in fostering positive perceptions and improving EHR usability among physicians. By evaluating this initiative in a real-world setting, the study contributes to the broader literature on strategies aimed at enhancing physician experience following large-scale EHR implementation. However, the findings indicate a continued need for system-level improvements to maximize the value and usage of EHRs. The physician engagement strategy demonstrates the potential to enhance physicians’ EHR experience. Future efforts should prioritize system-level advancements to increase the EHR’s impact on quality of care and develop standardized approaches for engaging physicians on a broader Canadian scale.

## Introduction

### Background

The burden caused by the use of electronic health record (EHR) systems continues to be an important issue for health care organizations, especially given human resource shortages in health care systems globally [1-4]. As physicians report spending 2 hours documenting for every hour of patient care, there has been strong interest from many organizations to understand and address the root causes of physician burnout due to EHR burden [5]. This is particularly important given the growing digital maturity of many health systems coupled with broader health care challenges, specifically an overburdened health system and a health care workforce crisis [2-4]. Over the last few years, many organizations, including the American Medical Informatics Association [6], the Canadian Medical Association [2-4], and the American Medical Association [7], have recognized and advocated for strategies to target these challenges .

As a result, there have been many initiatives focused on mitigating the impacts of EHR burden on physicians. For example, the 25 × 5 initiative, led by American Medical Informatics Association and including a task force representing health professionals or systems, policy or advocacy, impact, and technology requirements, is focused on developing a multi-level approach (vendor, health systems, and government) to reducing documentation burden by 25% in 5 years in the United States [6,8]. Through these work streams, a toolkit has been developed that provides recommendations and next steps at the organization and system levels [8]. In addition, others have explored unique initiatives to identify and address EHR-related burden within their own organizations [9]. An example of this is the Getting Rid of Stupid Stuff initiative from the University of Colorado, which focuses on removing redundant processes from the EHR [10]. Other initiatives include more agile delivery of personalized training and system optimization targeting the pain points of the end-users [11-13].

However, a key challenge that has impacted the ability to develop strong recommendations on approaches to address EHR-related issues has been limited insight into the impact of these initiatives [9,14,15]. In particular, most of these evaluations have been limited to a single-site pilot with a limited duration and small participant size. Moreover, since these studies examined the intervention immediately after implementation, there is little information about the long-term impact of these initiatives on EHR end-user experience [16]. To better understand the impact of initiatives to reduce EHR burden, it is essential to evaluate them in large-scale real-world environments with numerous users, as studies of digital tools indicate that effectiveness must be tested across diverse settings. Overall, this study focuses on evaluating physician burnout related to EHR usage and the impact of a physician engagement strategy at a Canadian mental health organization 5 years post-implementation.

### Physician Engagement Strategy

The physician engagement strategy [17] was developed in 2018 through feedback from an organizational benchmark survey, which was conducted to understand the contributors of EHR-related burden and burnout rate among physicians across a large Canadian academic hospital [18]. In the benchmark survey, full-time and part-time physicians at the organization were invited to provide their perceptions and concerns in order to co-develop the first iteration of the physician engagement survey. Briefly, from the benchmark survey, about 75% (155/208) of physicians with symptoms of burnout reported that the EHR was a major contributor to burnout. In addition, participants highlighted process and infrastructure issues that can be improved through an organization-wide strategy.

As such, the physician engagement strategy was created and implemented and consists of 4 key components.

#### The “SWAT” Initiative

One of the main challenges has been an approach to rapidly identify and address bottlenecks and issues related to the EHR. As a result, we developed the “SWAT” initiative, which focuses on bringing an interdisciplinary team to rapidly triage and address issues related to the EHR in an agile manner [19].

#### Physician Think Tanks

Given the ongoing challenges of ensuring that decisions made for the EHR are aligned with the needs of end-users, a medical staff subcommittee was convened to engage in discussions on EHR and digital health matters. These think tanks meet on a monthly basis, and a representative from each medical division attends to provide feedback and discuss relevant issues.

#### Communication, Education, and Informatics Strategies

In order to address the outstanding issues from the benchmark survey, numerous initiatives were implemented. Several peer education videos to provide peer support on common issues that physicians encounter in using the EHR. In addition, a monthly newsletter was implemented to communicate new changes that have been implemented to the EHR. Lastly, speech recognition technology (SRT) was procured and implemented to improve efficiency in documentation. SRT allows physicians to dictate their documentation directly into the EHR instead of typing [20].

#### Using EHR Usage Log Data to Guide Further Optimizations

In order to assess the impact of these interventions on the EHR, back-end usage log data are being collected and reviewed. The solution is included as part of the EHR system and includes metrics such as documentation time per patient and after-hours use of the EHR (pajama time).

While smaller evaluations have been conducted on individual components [19-21], there remains a gap in understanding how the overall strategy has supported improvements in EHR use over the past 5 years. It is vital to explore opportunities for continuously advancing the strategy as new technologies, such as generative artificial intelligence, are integrated into the environment [22-28]. This is particularly useful given the growing issue of physician burnout and the rapid growth of digital tool adoption in health care organizations outside of Canada [29-32]. The core contribution of this manuscript is to demonstrate the impact and lessons learned from implementing a multi-pronged organizational strategy focused on reducing EHR burnout in a real-world environment, as well as some of the outstanding areas of focus. This work helps support our limited understanding of these initiatives. This is particularly relevant to other countries and hospitals as they gain more advanced, comprehensive EHRs such as the ones being used at this hospital. EHR-related burnout is a global issue, and these initiatives can be translated to other organizations facing similar challenges.

### Objectives

In this study, we present our findings from a survey conducted 5 years after the implementation of the physician engagement strategy. The primary objective is to understand the impact and perceptions of the strategy’s 4 components and identify opportunities for further improvement. In order to do so, a survey was developed that asks questions related to the effectiveness of each of the components of the strategy. These findings may be valuable for Canadian health care organizations seeking to adopt a long-term strategy to enhance physician experience and reduce EHR-related burden.

The specific aims of this study were to evaluate overall burnout rates and the extent to which they are linked to the EHR 5 years after implementing the physician engagement strategy; assess the perceived effectiveness of the different components within the physician engagement strategy; and pinpoint key areas for future improvement and optimization of the physician engagement strategy to further alleviate EHR-related burden.

## Methods

### Overview

A cross-sectional survey was conducted in January 2023 for 6 weeks, to evaluate the impact and challenges in delivering the physician engagement strategy at a large, urban, Canadian mental health hospital.

### Participants and Settings

In order to identify physicians who are eligible for the survey, we obtained a list from the Medical Affairs department. The inclusion criteria for physicians are as follows: a staff physician, resident, or fellow at the organization having a full-time or part-time appointment at the organization as of November 2022, and having a psychiatry, addictions, or hospitalist role that provides care across inpatient, outpatient, emergency, outreach, and community settings.

Physicians who joined after November 2022 were not invited to participate in the survey due to their limited exposure and use of the EHR prior to completing the survey.

In terms of setting, the large academic mental health hospital is located in Toronto, Ontario, and provides care to over 34,000 patients a year. The EHR system was implemented in 2014 and is being used for all facets of patient care. In 2017, the organization achieved Healthcare Information Management Systems Society (HIMSS) stage 7 on the Electronic Medical Record Adoption Model (ENRAM) [33], which is used to identify gaps in EHR usage, guide digital transformation, improve data access and patient outcomes, and reduce grunt work. Achieving the highest level, stage 7, of this model is a testament to the high adoption and use of the EHR for clinical care and requires our organization to use our EHR in dynamic ways and leverage analytics and insights.

### Data Collection Approach

A web-based survey was deployed to physicians via the REDCap survey tool (developed by Vanderbily University) [34,35]. The survey was developed using a co-design approach with members of the Physician Think Tanks and was informed by the Benefits Evaluation Framework, benchmark survey data, and relevant literature. The survey underwent testing with the Physician Think Tank members to refine its topics, content, and wording. It was available from January to March 2023, with biweekly reminders sent to encourage participation. The Chief Medical Officer (SS) and the Division Chiefs encouraged physicians to participate in the survey. The survey has 4 components, which can be found in Multimedia Appendix 1. The first comprises demographics, which include age, gender, appointment, and years of experience in the organization. The second component focuses on burnout, which includes the Mini-Z Burnout questionnaire [36], as well as a question on the perceived contribution of the EHR to burnout. The third section contains questions related to the perceived impact of the initiatives within the physician engagement strategy, as well as emerging issues of interest outlined by the Physician Think Tanks. The last section is an open-ended question for participants to outline opportunities for improvement in terms of the use of the EHR and initiatives within the physician engagement strategy.

### Data Analysis

Both quantitative and qualitative approaches were used to analyze the data. For Likert scale questions, descriptive statistics (eg, mean, standard deviation) were generated. In addition, one-way ANOVA and Chi-squared tests were used to compare burnout rates from the benchmark survey. For qualitative data, a content analysis approach was used to identify the main themes [37,38]. The analysis was led by authors TT and BL. TT conducted a review of the qualitative responses and developed an initial codebook. BL then reviewed and identified any discrepancies. Any discrepancies were reviewed and discussed with the rest of the project team. Based on the revised codebook, TT completed the coding of the qualitative responses. BL then reviewed the coding to identify and discuss any additional discrepancies. To ensure the trustworthiness of the data, 2 reviewers have analyzed the data using the approaches outlined by Elo et al [39]

### Ethical Considerations

This work has received approval by the Quality Project Ethics Review (QPER) Board at the Centre for Addiction and Mental Health (QPER 2018‐2019 #014) as it was a quality improvement project and did not require approval from our Research Ethics Board. Informed consent was obtained for physicians participating in the project. Numerous safeguards were also implemented to ensure the confidentiality of participants. First, data were collected anonymously. Participants were instructed not to share identifying information in their responses. All survey data were de-identified before analysis if individuals entered identifying information in the survey. Second, findings were reported only in aggregate form to both internal and external audiences. Compensation was not provided to participants.

## Results

### Participants

Of 254 staff physicians eligible to complete the survey, 128 participants completed it and were included in the analysis (50%). There were 18 individuals who opened the link to the survey but did not submit the survey. While we had originally invited residents and staff fellows to the survey, despite our extensive efforts in recruitment, we had received very few responses resulting in a negligible response rate. Hence, they were not included in the analysis of the survey. The findings of the survey are described below.

### Respondent Characteristics

The demographics reported by participants who completed the survey (n=128) are outlined in Table 1. Respondents were primarily staff physicians with outpatient duties and provided psychiatric care to adult populations. There was a range in years of experience of respondents, with 27% (35/128) of participants, reporting 0‐5 years of experience. In terms of gender identity and ethnicity, there was about equal responses from participants identifying as a man (45%, 58/128) or woman (42%, 54/128), and 53% of individuals (68/128) identified with a white ethnic group.

**Table 1.** Demographic characteristics of respondents.

Characteristic category	Number of participants (%) (n=128)
Clinical activities (multiple options were allowed to be selected)	
Inpatient physician	46 (36)
Outpatient physician	93 (73)
Emergency department	22 (17)
Community-based care	10 (8)
Outreach	8 (6)
Primary division	
Addictions	23 (18)
Adult neurodevelopment and geriatric psychiatry	12 (9)
Child and youth psychiatry	11 (9)
Forensics	8 (6)
General adult and health systems psychiatry	46 (36)
Hospital medicine	15 (11)
Schizophrenia	13 (10)
Frequency in providing care	
Full-time (≥0.6FTE)	106 (83)
Part-time (<0.6FTE)	22 (17)
Experience in practicing as a physician	
0‐5 Years	35 (27)
6‐10 Years	26 (20)
11‐15 Years	24 (19)
16‐20 Years	12 (9)
21‐25 Years	12 (9)
26+ Years	18 (14)
Prefer not to answer	1 (1)
Age group	
<30 years old	3 (2)
31‐40 years old	46 (36)
41‐50 years old	39 (30)
51‐60 years old	19 (15)
61+ years old	13 (10)
Prefer not to answer or did not respond	8 (6)
Gender identity	
Man	58 (45)
Non-binary	2 (2)
Woman	54 (42)
Prefer not to answer or not reported	14 (11)
Ethnicity	
East Asian	11 (9)
Latin American	2 (2)
Middle Eastern	9 (7)
South Asian	10 (8)
Southeast Asian	2 (2)
White	68 (53)
Indian-Caribbean	1 (1)
Indigenous	1 (1)
Mixed ethnicity	4 (3)
Identity not listed	2 (2)
Prefer not to answer or did not respond	18 (14)

### Burnout and Perceptions of the EHR

The level of burnout reported by participants using the Mini Z questionnaire [32] is outlined in Table 2. About 26% (33/128) of individuals reported a description that meets the threshold of burnout. However, only 4% (5/128) of individuals reported being at the highest and second-highest levels of burnout. Of those who indicated a level that meets the threshold of burnout, 61% (20/33) of the respondents indicated that the EHR contributed to their burnout often or always. There was no relationship between burnout levels and age, ethnicity, or years of experience.

**Table 2.** Reported levels of burnout for benchmark survey and follow-up survey.

		Burnout, n (%)	
Characteristic category		Baseline survey (N=208) [18]	Follow-up survey (N=128)
I enjoy my work. I have no symptoms of burnout.		45 (22)	27 (21)
Occasionally I am under stress, and I don’t always have as much energy as I once did, but I don’t feel burned out		111 (53)	68 (53)
I am definitely burning out and have one or more symptoms of burnout, such as physical and emotional exhaustion		35 (17)	28 (22)
The symptoms of burnout that I’m experiencing won’t go away. I think about frustration at work a lot.		15 (7)	4 (3)
I feel completely burned out and often wonder if I can go on. I am at the point where I may need some changes or may need to seek some sort of help.		1 (1)	1 (1)

There were no significant differences in burnout level (t(283)=.191, *P=.*85) between the current survey and the previously conducted benchmark survey [18]. Of those who reported burnout, there is a considerable reduction in the number of individuals who reported that the EHR contributes to burnout all or almost all the time (*t*(63)=−1.255, *P*=.21).

With regard to the perceptions towards the EHR, over half the participants (74/128) agreed or strongly agreed that the EHR adds to their daily frustration (Table 3). From a benefits perspective, a similar proportion of participants also felt that the EHR improved communication within their circle of care (67/128), enabled the delivery of high-quality care (49/128), and kept patients safe (50/128).

**Table 3.** Perceived effectiveness of the various components of the physician engagement strategy.

Characteristic category	Participants, N=128, n (%)
Do you think [EHR]^a^ contributes to your symptoms of burnout? (only for those who answered having at least one or more symptoms of burnout (n=33))	
Rarely	1 (3)
Sometimes	12 (36)
Often	14 (42)
Always	6 (18)
[EHR] adds to my daily frustration.	
Strongly disagree	2 (2)
Disagree	20 (16)
Neutral	32 (25)
Agree	51 (40)
Strongly agree	23 (18)
[EHR] improves communication within the circle of care.	
Strongly disagree	2 (2)
Disagree	25 (20)
Neutral	34 (27)
Agree	55 (43)
Strongly agree	12 (9)
[EHR] enables me to deliver high-quality care.	
Strongly disagree	8 (6)
Disagree	18 (14)
Neutral	53 (41)
Agree	43 (34)
Strongly agree	6 (5)
[EHR] helps keep my patients safe.	
Strongly disagree	4 (3)
Disagree	19 (15)
Neutral	55 (43)
Agree	44 (34)
Strongly agree	6 (5)

a EHR: electronic health record.

### SWAT Initiative—Rapid Handling of EHR Issues

**Figure 1.**
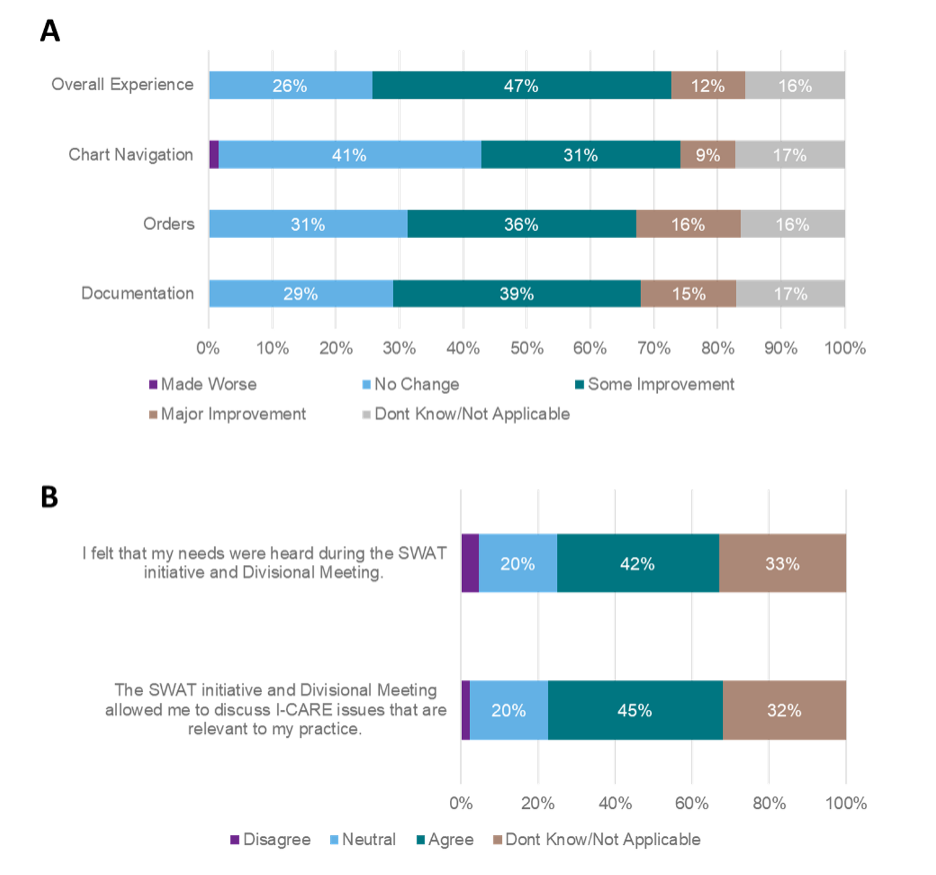
Impact of SWAT (agile approach for resolving electronic health record (EHR)-related issues) initiative on EHR burden (n=128).

In the survey, participants indicated whether the SWAT initiative improved documentation, order entry, and chart navigation within the EHR (Figure 1A). Of those who provided a response, about 54% of participants reported that the SWAT initiative had made some or major improvements across documentation (69/128) and order entry. For chart navigation, the numbers of respondents were marginally less, with 40% of participants indicating that SWAT has made some or major improvements to the functionalities related to chart navigation (51/128).

In terms of overall experience (Figure 1A), approximately 59% (75/128) of respondents reported that the SWAT initiative has made some or major improvement to their use of the EHR. Similarly, about 42% (54/128) of participants agreed or strongly agreed that the initiative made them feel their needs were acknowledged and 45% (58/128) agreed that it provided a platform to discuss issues pertinent to their practice (Figure 1B).

### Communication and Education

**Figure 2.**
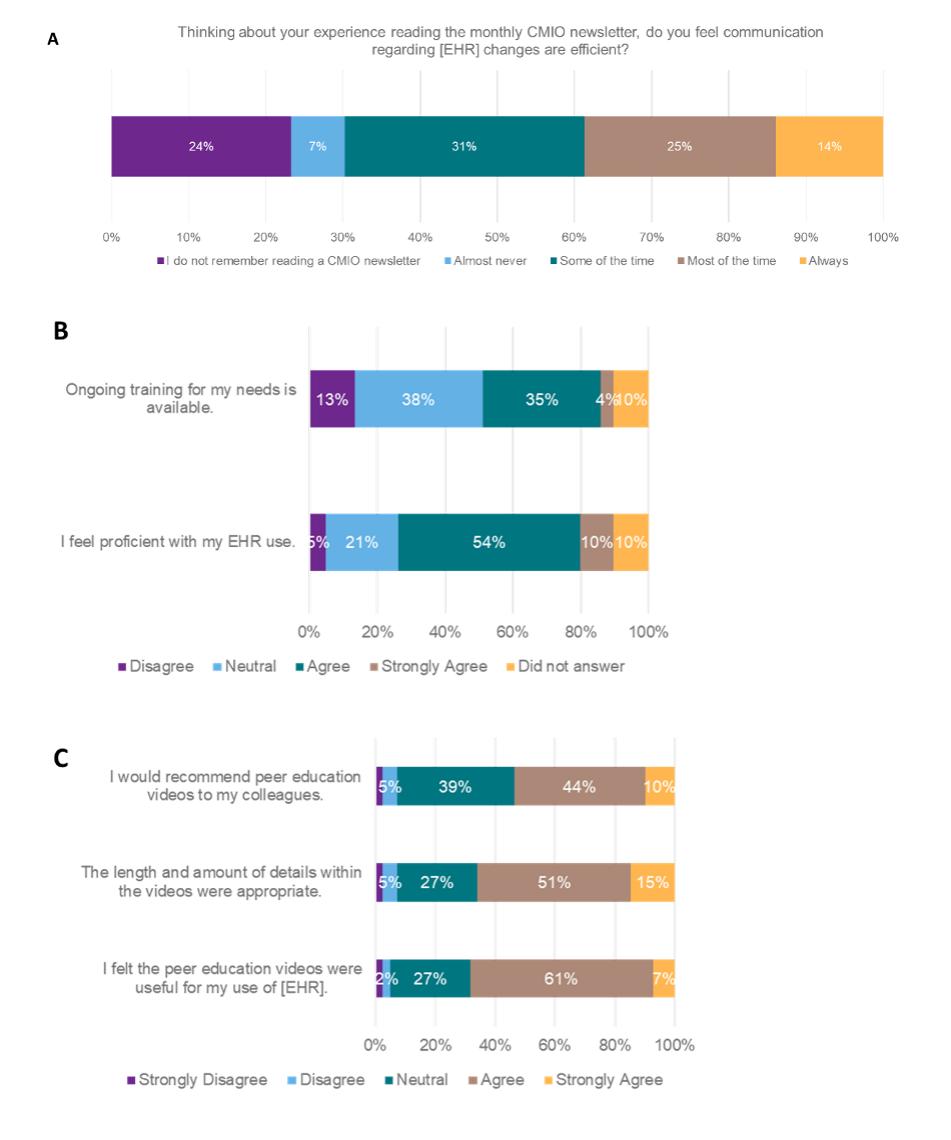
Impact of communication and education initiatives (n=128). EHR: electronic health record.

With regard to communication, about 77% (98/128) of respondents recall reading the monthly newsletter that is sent out from the strategy (Figure 2A). Of those who have read the newsletter, about half (50/98) suggested that the communication of EHR changes is efficient through this method.

For education, about 64% (82/128) of individuals believe they are proficient with the use of the EHR, yet only 39% (50/128) of individuals thought that the education support provided by the organization is available and sufficient (Figure 2B). Regarding peer education videos (Figure 2C), about 32% (41/128) of individuals reported watching 1 of the 3 peer education videos released to date. For those who did not watch a video, about 57% (73/128) of respondents were not aware that peer education videos existed. Among the 41 individuals who watched a video, 68% (28/41) of individuals reported that the video was useful, and 66% (27/41) of individuals believed that the length and details were appropriate.

### Informatics Tools (SRT Documentation and Continuity of Care)

**Figure 3.**
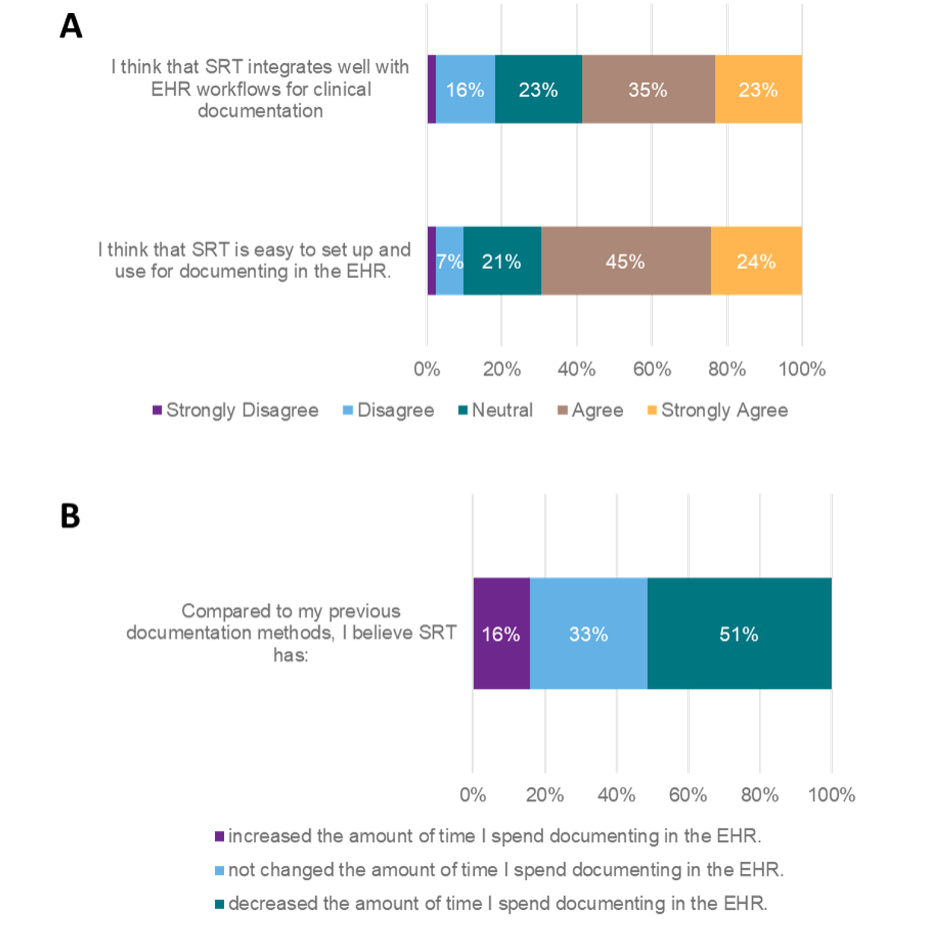
Impact of speech recognition technology on documentation in the electronic health record (EHR; N=128). SRT: speech recognition technology.

With regard to documentation, about 64% (82/128) of respondents reported trying to use SRT for documentation (Figure 3A). Of those who indicated using SRT, 69% (57/82) of individuals reported that it was easy to set up and use, and 58% (48/82) of respondents indicated that it integrates well with the EHR (Figure 3). About 51% (42/82) of respondents believed that the use of SRT decreased their documentation time (Figure 3B).

**Figure 4.**
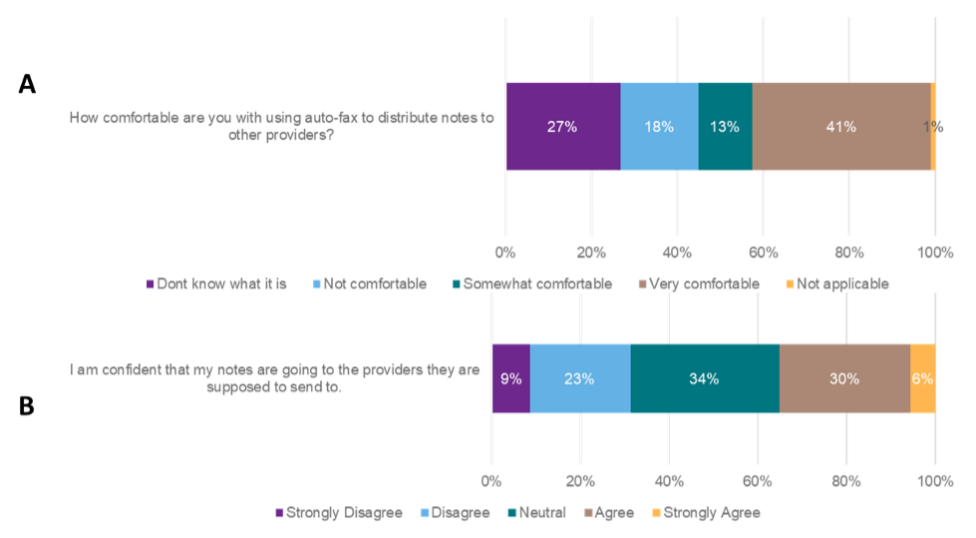
Perceptions of continuity of care in the electronic health record (n=128).

For continuity of care, participants were asked to comment on their confidence level regarding delivery of notes to external health care providers. Only 35% (45/128) of individuals agreed that they are confident that notes are sent to the provider as intended (Figure 4A). With automated fax transmission being the default approach for document delivery within the EHR, 54% (69/128) of respondents indicated that they are somewhat or very comfortable with sending their notes through the current automated fax transmission process instead of sending notes through health records distribution process (Figure 4B).

### Areas of Focus

From the qualitative findings, 4 themes were identified. These include (1) medication reconciliation and prescriptions processes; (2) chart navigation and information retrieval; (3) longitudinal medication history; and (4) technology infrastructure challenges. These themes are detailed below.

#### Medication Reconciliation and Prescriptions Processes

Physicians had concerns about the current capabilities of the EHR with regard to medication reconciliation and transmission of prescriptions. While medication reconciliation and communication of prescriptions were identified as highly important for supporting patient care, the processes involved were identified as cumbersome and time-consuming. As one physician outlines:

Outdated and clunky interface - navigation is clumsy and could be streamlined. More like 2010 than 2023 software…Med Reconciliation is a slow and laborious chore - great when pharmacists do it.

[Psychiatrist, SC-FT-246]

Another physician also highlights the lack of intuitive search functionality with the current EHR system:

Discharge med rec is frequently broken and unsafe. Search function is desperately needed. For example, cognitive testing hard to find or not accessible, not organized, not uploaded.

[Geriatrics Psychiatry Physician, GE-APP-47]

Collectively, these limitations of the system can make the process unsafe for patient care. Furthermore, the impact on continuity of care back to the community also affects the process at discharge. Fax is still commonly used for transmission of prescriptions and documents to community providers and pharmacies, but the limitations of this technology represent a significant burden for clinicians. This is particularly outlined from a physician who engages in telepsychiatry practice:

*The prescription function on [the EHR] is awful for telemedicine prescriptions. First, I have to enter the prescription on the software. Next, I have to download the PDF, find that, and sign manually. Finally, I have to open separate software [company] to fax the prescription. This whole process takes 5‐10 minutes, whereas with the other EMR software I can send a prescription in<1 min. Since I send many prescriptions per day, I can waste anywhere between 30‐60 min of a clinical day on this cumbersome prescription faxing process.*

[Telemedicine Psychiatrist, AD-AP-22]

Collectively, the administrative burden spent on faxing prescriptions can take a significant toll on physicians. More importantly, the lack of feedback from the process hinders the ability of the physician to know if the process worked for the patient:

*Sending external lab requisitions and prescriptions, especially when delivering virtual care is non-obvious, tedious, time-consuming and error prone. For instance, for nearly 3 months my prescriptions had a return fax number that no one could tell me if and where returned faxes from pharmacies were going to.*

[Addiction Psychiatrist, AP-AD-6]

Given the growing adoption of team-based care within the community, continuity of care through current medication reconciliation processes and faxing significantly hinders the value and utility of the EHR.

#### Chart Navigation and Information Retrieval

Given the complexity and chronicity of the patient population, chart navigation was another critical theme outlined by physicians. For example, several physicians highlighted issues about being able to view other notes while documenting:

*It is horrific that one cannot simultaneously see a document and type into a note. I don’t know who designed such a system, they do not understand physician work at all. Opening an ED note locks you out of seeing other documentation.*

[Addiction Psychiatrist, AD-FT-33]

*Being unable to quickly click through the pages, for example have a tab to click one month back or to be able to skip an inpatient admission. Being unable to have a note open while I am also looking at other applications. Have to write the draft note somewhere else and paste it.* [General Adult Psychiatrist, GA-FT-171]

As such, being able to review and generate new documentation can be burdensome and can result in developing workarounds that are not aligned with best practices. Another key limitation of the system is the limited ability to search for relevant documentation. Given that some patients have many previous consultations, searching through prior documentation efficiently is critical. One physician highlighted that it:

*It can be hard to find needed documentation if the chart is extensive because the filtering system isn’t great/doesn’t let you just search for the things you need.*

[General Adult Psychiatrist, GA-FT-158]

As such, this can hinder the ability for physicians to understand the broader clinical picture in a timely manner through the EHR.

#### Longitudinal Medication History

A review of past medication history often informs decision-making regarding which medication regimen to trial next for a particular patient. However, currently, there is a limited ability to review the medication histories of patients in a longitudinal way:

*[The EHR] is not well set up for longitudinal care. Medication histories, for example, are not well captured, nor are key pieces of information easily parked somewhere that can be referenced later without having to scroll through many progress notes.*

[Schizophrenia Physician, SZ-AP-220]

Having to review numerous progress notes can make it difficult for physicians to grasp the patient’s history and develop an evidence-based treatment plan.

#### Challenges with Technology Infrastructure

Since the implementation of the EHR 10 years ago, physicians have reported growing issues with regard to the challenges with IT infrastructure. For example, one psychiatrist highlights how the application slows down the system when open and takes excessive amounts of time to complete tasks within the EHR.

*The program freezes frequently and then it is a process to restart it. [The EHR] often has a lag and this slows down work for outpatient work, there is no easy way to fax prescriptions. What I do is create a PDF, open it in program (Premium which I pay for personally), sign it, save it, then open the [fax] program, and then send it. The fax process itself takes 5+ minutesand isn’t an effective use of physician time.*

[General Adult Psychiatrist, GA-FT-184]

This process becomes especially challenging when physicians must act under pressure, such as during an emergency code:

*To work on multiple units when on call- Duty Doctor is very time consuming as clinicians are required to start up their [EHR] on a new computer on each unit and the initializing process to get into the platform to access [EHR] takes 5‐10 minutes sometimes which is very delayed when responding to a code where a clinician is required to order meds/restraints etc.*

[General Adult Psychiatrist, GA-FT-114]

Since IT infrastructure is fundamental to EHR usability, it is crucial that it remain up to date to ensure the system operates efficiently and effectively over time, particularly in critical situations.

## Discussion

### Principal Findings and Comparison With Previous Work

This study examines the current findings from the physician engagement strategy at a Canadian mental health hospital in Toronto, Canada. Although extensive literature has investigated the causes of EHR-related physician burnout and proposed various approaches to address these issues [6], there is limited research on how physician engagement strategies and their components interact strategically to mitigate burnout through a health informatics perspective. To our knowledge, this work is among the first to involve a large number of Canadian physicians in assessing the impact of these initiatives within a cohesive and supportive environment. The findings from this work are critical given that many of the current studies are pilot or feasibility studies in a small setting or clinic. This work provides preliminary insights into how these interventions can be expanded to an organization and their impact. This is particularly important given the growing adoption of advanced EHRs with decision support, artificial intelligence, and other capabilities [40-44]. Overall, the physician engagement strategy has positively impacted perceptions and value of the EHR among physicians. However, there are still many opportunities for improvement, particularly at the system level. The implications of these findings are described below.

We found that while the physician overall burnout rate remained unchanged from the benchmark survey in 2018, the perceptions of the EHR’s impact on physician burnout have considerably improved. Physicians who used the physician engagement initiatives reported enhancements in EHR usage, communication, education, and documentation, which are key components of the overall strategy [18]. These findings are consistent with the growing literature on the role of perceptions of physician burnout. Several studies have highlighted how perceptions can influence overall satisfaction and utility of the EHR, despite its impact from a quantitative (objective) perspective [45]. Studies that take a sociotechnical perspective suggest that characteristics of the individual and the environment can have a profound impact on burnout rate and EHR usage patterns [46]. With this in mind, it is likely that understanding EHR-related burnout requires a broader consideration of the environment and the system at play.

Since the evaluation was conducted 5 years post-implementation of the strategy, improvements seem to be sustained over time. This provides promising support for the long-term impact of initiatives such as a physician engagement strategy, which is an area that has not been widely investigated [6].

Several areas for improvement have been identified since the strategy’s inception, many of which necessitate system-level interventions. For example, through the qualitative SWAT interviews, issues related to the transmission of prescriptions and documentation continue to be critical EHR-related barriers to continuity of care. In the Canadian setting, initiatives supporting electronic prescribing continue to progress at a slow pace, despite the high interest and discussion over the last decade [47]. In addition, functional interoperability could be used to support better continuity of care and the sunsetting of aging technology such as faxing [48]. As such, while these initiatives have made improvements to the use of the EHR, there continues to be a significant requirement for novel, system-level solutions that fall outside the scope of a single organization or single digital tool. As a next step from this work, the project team will focus the efforts of the strategy on addressing these challenges.

Finally, this work makes a significant contribution to the international literature on interventions to reduce EHR burden for physicians. To date, most of the literature, including the 25 × 5 toolkit and the Getting Rid of Stupid Stuff initiative, has a strong focus on the US context [10]. However, these constraints do not necessarily apply to the Canadian and international context. As such, the impact observed in this study provides suggestive evidence of the broad impact these tools can have, irrespective of clinical and administrative environments. Future work should focus on examining the impact of similar initiatives and strategies in other geographical and cultural contexts.

This work has broad-reaching implications for health care organizations and health systems. At the organization level, clinicians and health administrators can consider building and evaluating similar strategies within their own organizations. By leveraging similar evaluation approaches and methodologies (eg, Mini-Z for burnout), it will enable comparison and optimization of the initiatives outlined in this strategy. For researchers, it would be useful to explore how theoretical frameworks from implementation science and change management can be used to further understand the impact and key success factors in rolling out these initiatives across complex health care environments. At the health policy and system level, policy makers and health care organizations can leverage the findings from this study to inform the development of system-level recommendations and initiatives to address the root causes that are consistent across the health care system (eg, interoperability).

### Limitations

The study’s findings should be interpreted with caution due to several limitations. Foremost, the strategy is currently being evaluated in a single health care organization for staff physicians only. Despite our efforts to recruit residents and fellows through engagement of leadership and communications, the rapid turnover and limited time at the organization make it difficult to recruit learners to complete the survey. Future work should focus on exploring ways to enhance reach and engage these groups in EHR-related burden. Moreover, due to the limited number of time points available in the evaluation, this study fails to examine how the broader environment and the temporal nature of events can influence the impact of this strategy on EHR burden. In addition, there are currently limited validated instruments to examine the effectiveness of these interventions. As such, the questions of the survey were based on the feedback from the liaison members of the Physician Think Tank and the members of the project team. Future work should focus on developing validated survey instruments to assess initiatives for reducing EHR-related burden [6]. Lastly, this study did not examine objective data sources like EHR usage logs, which could provide a more comprehensive understanding of the initiatives’ impacts.

### Conclusions

This study evaluates the impact of a physician engagement strategy on improving physician experience and reducing EHR burden at a Canadian mental health organization. The study highlights the ongoing challenges and perceptions of staff physicians regarding the EHR system, emphasizing its impact on burnout, workflow efficiency, and patient care. While physicians recognized that the EHR improved communication and supported high-quality care, the system also played a significant role in daily frustration for many physicians, with many citing the usability of the system as a major challenge. However, the cross-sectional survey suggests significant improvements in EHR use since the inception of the strategy in 2018. In addition, the findings from the survey demonstrate the feasibility and preliminary effectiveness of these initiatives on reducing EHR burden at an organizational level. Despite some positive findings, additional interventions and system-level changes are needed to further reduce EHR burden and enhance physician experience and patient care. Gaps continue to persist in areas such as medication reconciliation, chart navigation, and the reliability of IT infrastructure. Targeted improvements such as streamlining prescription processes, enhancing search functionalities, and addressing system inefficiencies could alleviate some administrative burdens and improve physician satisfaction. Addressing these concerns is essential to optimizing the usability of the EHR and supporting physician well-being in clinical practice. Future work should also focus on exploring how these system-level changes can be adopted and explored in other health systems.

## Supplementary material

10.2196/65656 Multimedia Appendix 1 Physician Survey.

## Abbreviations

EHR: electronic health record
QPER: Quality Project Ethics Review
SRT: speech recognition technology
SWAT: agile approach for resolving EHR-related issues
