# Supplementary material for: Assessing the Impact on Electronic Health Record Burden After Five Years of Physician Engagement in a Canadian Mental Health Organization: Mixed-Methods Study
Source: JMIR Hum Factors. 2025 May 9;12:e65656. doi: 10.2196/65656 (PMC12083741; doi:10.2196/65656)
Supplement: Multimedia Appendix 1 [file humanfactors-v12-e65656-s001.pdf]

Physician I-CARE Survey

In 2019, you completed the I-CARE Benchmark Survey which was used to formulate our Physician Engagement Strategy - thank you for your input. Since that time, we have spearheaded a number of efforts to improve the I-CARE experience for our physicians, residents and fellows by:

Addressing over 130 issues as part of the SWAT initiative;Discussing over 70 topics with I-CARE Liasions through the Physician Think Tank;Releasing 5 peer education videos;Publishing 40+ CMIO newsletters with updates and changes about I-CARE;Rolling out DMO Speech Recognition Technology to our physicians/residents/fellows (with over 1.7 million lines of dictation completed in 2022)As we plan out what's next for our Physician Engagement Strategy, we would like to request 5-10 minutes of your time to complete the below survey to tell us about your experience with I-CARE, your experience with the initiatives of the strategy and what you think we should focus next for I-CARE.

Note that the survey has been reviewed by the CAMH QPER Committee and your responses are anonymous and will only be shared in aggregate with the project team and in publications/presentations. Your decision to complete this survey is completely voluntary and will not impact your professional relationship with CAMH. If you would not like to answer a demographic or survey question, feel free to skip it.

Your feedback would be greatly appreciated. If you have any questions, please reach out to Brian Lo (Project Lead) at Brian.Lo@camh.ca

Demographics

I am a:	◯ Staff physician<br>◯ Fellow<br>◯ Resident
How would you describe your clinical activities?	☐ Inpatient Physician<br>☐ Outpatient Physician<br>☐ Emergency Department<br>☐ Community-based care (e.g., ACT, integrated care within primary care settings)<br>☐ Outreach (e.g., Northern Psychiatric Outreach Program at CAMH(NPOC-C))<br>(Please select all that apply.)
What is your primary division?	◯ Addictions<br>◯ Adult Neurodevelopment and Geriatric Psychiatry<br>◯ Child and Youth Psychiatry<br>◯ Forensics<br>◯ General Adult and Health Systems Psychiatry<br>◯ Hospital Medicine<br>◯ Schizophrenia
How frequently do you provide clinical care at CAMH?	◯ Part-time (< 0.6 FTE)<br>◯ Full-time (≥ 0.6FTE)
How long have you been practicing as a physician?	◯ 0-5 years<br>◯ 6-10 years<br>◯ 11-15 years<br>◯ 16-20 years<br>◯ 21-25 years<br>◯ 26+ years<br>◯ Prefer not to answer
What is your age group?	◯ < 30<br>◯ 31-40<br>◯ 41-50<br>◯ 51-60<br>◯ 61+<br>◯ Prefer not to answer

Please select the option that best describes you:	◯ Man<br>◯ Non-Binary<br>◯ Questioning<br>◯ Two-spirit<br>◯ Trans Man<br>◯ Trans Woman<br>◯ Woman<br>◯ Identity not listed (enter below)<br>◯ More than one gender identity<br>◯ Prefer not to answer
Gender_other	__________________________________<br>(df)
Which of the following best describes your racial or ethnic group?	◯ Black African<br>◯ Black Caribbean<br>◯ Black North American<br>◯ East Asian<br>◯ First Nations<br>◯ Indian-Caribbean<br>◯ Indigenous<br>◯ Inuit<br>◯ Latin American<br>◯ Metis<br>◯ Middle Eastern<br>◯ South Asian<br>◯ Southeast Asian<br>◯ White<br>◯ Mixed Ethnicity<br>◯ Identity not listed (enter below)<br>◯ Prefer not to answer
Ethnicity_other	__________________________________

Use of I-CARE

Using your own definition of burnout, select one of the answers:

- ◯ I enjoy my work. I have no symptoms of burnout.
- ◯ I am under stress, and don't always have as much energy as I did, but I don't feel burned out.
- ◯ I am definitely burning out and have one or more symptoms of burnout, e.g., emotional exhaustion.
- ◯ The symptoms of burnout that I am experiencing won't go away. I think about work frustrations a lot.
- ◯ I feel completely burned out. I am at the point where I may need to seek help.

Do you think I-CARE contributes to your symptoms of burnout?

- ◯ Always
- ◯ Often
- ◯ Sometimes
- ◯ Rarely
- ◯ Never

Please rate each statement from strongly disagree to strongly agree.

	Strongly Disagree	Disagree	Neutral	Agree	Strongly Agree
I-CARE adds to my daily frustration.	◯	◯	◯	◯	◯
I-CARE improves communication within the circle of care.	◯	◯	◯	◯	◯
I-CARE enables me to deliver high quality care.	◯	◯	◯	◯	◯
I-CARE helps keep my patients safe.	◯	◯	◯	◯	◯

Please let us know any challenges around using I-CARE that contributes to frustration.

____

How has your experience with I-CARE changed in the shift to more virtual/remote work?

____

What improvements have you seen in other EHRs that have facilitated your work elsewhere that you would like to see integrated here?

____

The SWAT Initiative and Divisional Meeting

In 2019-2020, we conducted a SWAT initiative (click here to see paper) with each division to identify and address high priority issues within the EHR. We started with over 130 changes and only 8 changes are left to date. Some of the changes made and education provided within your division from SWAT include:

[list of issues relevant to their divison] In 2022, we also came to your divisional meeting to discuss your top divisional priorities and I-CARE issues.

Thinking about all the collective changes and education from SWAT and the Divisional Meeting, how much has it improved your experience of I-CARE for

	Made Worse	No Change	Some Improvement	Major Improvement	Don't Know/Not Applicable
Documentation	◯	◯	◯	◯	◯
Orders	◯	◯	◯	◯	◯
Chart Navigation	◯	◯	◯	◯	◯
Overall Experience with I-CARE	◯	◯	◯	◯	◯

	Strongly Disagree	Disagree	Neutral	Agree	Strongly Agree	Don't Know/Not Applicable
The SWAT initiative and Divisional Meeting allowed me to discuss I-CARE issues that are relevant to my practice.	◯	◯	◯	◯	◯	◯
I felt that my needs were heard during the SWAT initiative and Divisional Meeting.	◯	◯	◯	◯	◯	◯

Please let us know how we can improve our SWAT and/or divisional meetings.

____

Communication & Education

Thinking about your experience reading the monthly CMIO newsletter, do you feel communication regarding I-CARE changes are efficient?

- ◯ Always
- ◯ Most of the time
- ◯ Some of the time
- ◯ Almost never
- ◯ I do not remember reading a CMIO newsletter

How can we improve our CMIO newsletters and/or communication around I-CARE to better meet your needs?

____

Education
Please rate each statement from strongly disagree to strongly agree.

	Strongly Disagree	Disagree	Neutral	Agree	Strongly Agree
I feel proficient with my I-CARE use.	◯	◯	◯	◯	◯
My initial training at CAMH prepared me to use I-CARE well.	◯	◯	◯	◯	◯
Ongoing training for my needs is available.	◯	◯	◯	◯	◯

Over the last two years, we released several peer educational videos (Medication Reconciliation, discharge summaries and speech recognition). Do you remember watching a peer education video?

- ◯ Yes
- ◯ No

Were you aware about the peer education videos?

- ◯ Yes
- ◯ No

Thinking about the peer education videos you watched, how much do you agree with the following statements?

	Strongly Disagree	Disagree	Neutral	Agree	Strongly Agree
I felt the peer education videos were useful for my use of I-CARE.	◯	◯	◯	◯	◯
The length and amount of details within the videos were appropriate.	◯	◯	◯	◯	◯
I would recommend peer education videos to my colleagues.	◯	◯	◯	◯	◯

Please let us know if you have any future ideas for Peer Education Videos or ways we can improve the delivery of I-CARE Education at CAMH.

____

Documentation and Continuity of Care

Have you tried Dragon Medical One (DMO) for your Documentation?
- ◯ Yes
- ◯ No

Please let us know any challenges that prevented you from trying DMO for documentation.

____

Thinking about your use of DMO in the last 3 months, rate the following statements:

	Strongly Disagree	Disagree	Neutral	Agree	Strongly Agree
I think that DMO is easy to set up and use for documenting in I-CARE	◯	◯	◯	◯	◯
I think that DMO integrates well with I-CARE workflows for clinical documentation	◯	◯	◯	◯	◯

Compared to my previous documentation methods, I believe DMO has:
- ◯ increased the amount of time I spend documenting in I-CARE
- ◯ decreased the amount of time I spend documenting in I-CARE
- ◯ not changed the amount of time I spend documenting in I-CARE

Please let us know if there are any issues or challenges with using DMO.

____

Continuity of Care: Auto-Fax and Document Distribution

One critical area that has been identified is continuity of care and distribution of notes to other providers using auto-fax. Auto-fax is the selection of providers before signing the note which appears as the cc field in the finalized note. In some cases, where the physician's name does not appear in I-CARE, physicians are asked to use the health records distribution function. We want to ask you a few questions about your use of this feature to share notes to external providers.

How comfortable are you with using auto-fax to distribute notes to other providers?
- ◯ Don't know what it is
- ◯ Not comfortable
- ◯ Somewhat comfortable
- ◯ Very comfortable
- ◯ Not applicable

What percentage of your notes are you sending through the auto-fax function?
- ◯ None of my notes
- ◯ Less than 50% of my notes
- ◯ More than 50% of my notes
- ◯ All of my notes
- ◯ I don't know

For scenarios where the auto fax function does not work, how often do you send your notes to external care providers through the health records distribution function?
- ◯ None of my notes
- ◯ Less than 50% of my notes
- ◯ More than 50% of my notes
- ◯ All of my notes
- ◯ I don't know

I am confident that my notes are going to the providers they are supposed to send to.	◯ Strongly Disagree<br>◯ Disagree<br>◯ Neutral<br>◯ Agree<br>◯ Strongly Agree
Please let us know if you have any suggestions for improving communication with external providers and/or comments about auto fax/distribution through health records.	__________________________________
